# Therapeutic challenges in two adolescent male patients with Fabry disease and high antibody titres

**DOI:** 10.1016/j.ymgmr.2020.100618

**Published:** 2020-06-24

**Authors:** Aizeddin A. Mhanni, Christiane Auray-Blais, Michel Boutin, Alie Johnston, Kaye LeMoine, Jill Patterson, Johannes M.F.G. Aerts, Michael L. West, Cheryl Rockman-Greenberg

**Affiliations:** a Department of Pediatrics and Child Health, Rady Faculty of Health Sciences, University of Manitoba, Winnipeg, MB, Canada; b Children's Hospital Research Institute of Manitoba, Winnipeg, MB, Canada; c Division of Medical Genetics, Department of Pediatrics, Centre de Recherche-CHUS, Faculty of Medicine and Health Sciences, Université de Sherbrooke, Sherbrooke, QC, Canada; d Nova Scotia Health Authority, Halifax, Nova Scotia, Canada; e Department of Medical Biochemistry, Leiden University, Leiden, the Netherlands; f Department of Medicine, Dalhousie University, Halifax, NS, Canada

**Keywords:** Fabry disease, Agalsidase antibodies, Enzyme replacement therapy, Globotriaosylceramide (Gb_3_), Globotriaosylsphingosine (Lyso-Gb_3_)

## Abstract

Enzyme replacement therapy (ERT) has been shown to stabilize certain aspects of Fabry disease (FD). However, in some patients on ERT, high antibody titres have been documented, with limited clinical improvement in systemic manifestations and often with significant adverse drug reactions. We present two related adolescent males with a 4.5 kb *GLA* deletion, not amenable to chaperone therapy, leading to profound reduction in α-galactosidase A (α-gal A) enzyme activity. Over a 3-year period of ERT, increasing IgG antibody titres against α-gal A were noted. After starting ERT serial urine globotriaosylceramide (Gb_3_) measurements showed an upward trend from 333 to 2260 μg/mmol creatinine for patient 1 and 1165 to 2260 μg/mmol creatinine for patient 2. Markedly increased levels of urine and plasma globotriaosylsphingosine (Lyso-Gb_3)_ analogues were also found. The patients experienced recurrent infusion-associated reactions necessitating premedication and prolonged infusion times. Over the 3-year period of ERT, the patients experienced continued malaise, gastrointestinal symptoms and neuropathic pain. In addition, they had increasing anxiety related to their disease and apparent lack of response to ERT which led to a decision to ultimately stop ERT. No other approved treatment options are currently available for these patients. It is possible that the rapid development of the high antidrug neutralizing antibody (ADA) titres is related to the large *GLA* deletion leading to virtually absent enzyme activity. It remains unclear if their symptomatology during the period of receiving ERT is related to lack of its efficacy, the rising ADA titres, or both. These two patients highlight the need for further research into the management of antidrug antibodies and additional therapeutic approaches for FD.

**Synopsis:**

The development of very high antidrug antibody titres in response to ERT in two related adolescent males with FD highlight the need for other therapeutic options for patients in whom ERT or other currently approved therapies does not meet their treatment needs.

## 1 Introduction

Fabry disease (FD) (OMIM #301500) is an X-linked multisystem lysosomal storage disorder (LSD) caused by a deficiency of α-galactosidase A enzyme (α-gal A, EC 3.2.1.22) also known as (ceramide trihexosidase). Several treatment modalities are currently approved including enzyme replacement therapy (ERT) in the forms of agalsidase alfa (0.2 mg/kg intravenous (IV) every 2 weeks, Replagal™, Shire/Takeda Pharmaceutical, Lexington, MA, USA) and agalsidase-beta (1.0 mg/kg IV every 2 weeks, Fabrazyme®, Sanofi Genzyme, Cambridge, MA, USA) and chaperone therapy with migalastat (123 mg capsule taken orally every other day, Galafold™, Amicus Therapeutics, Cranbury, NJ, USA) [20]. ERT has been shown to stabilize disease progression of the nephropathy and the cardiomyopathy and reduces gastrointestinal symptoms such as diarrhea, nausea, cramping, vomiting and heartburn [16]. In Canada, ERT treatment indications are based on the most recent guidelines published by the Canadian Fabry Disease Initiative (CFDI) (now CFDI-National Registry [NR]) (Sirrs et al. 2019, [36]) and these include significant renal disease, cardiac disease and/or neurologic disease. Gastrointestinal symptoms (GI) can be an indication to treat provided that symptoms are affecting quality of life or growth and have been unresponsive to other treatments for six months or greater. Neuropathic pain unresponsive to other measures as the sole indication for ERT can be tried for a year recognizing that response is minimal in many patients. ELISA assays have been used by ERT manufacturers who have identified IgG antidrug antibodies in many patients [7,33,39]. These antibodies are more frequent in males than females and in more patients receiving agalsidase beta than agalsidase alfa and associated with higher urine Gb_3_ and plasma Lyso-Gb_3_ levels suggesting reduced action of α-gal A as reviewed by [14]. Neutralizing antidrug antibodies to ERT in Fabry disease were first reported in 2004. The neutralization was identified in vitro with the highest published antibody titre to agalsidase alfa or beta of 1/32768 [27]. The association of agal-A antidrug antibody (ADA) with worsening clinical outcomes in FD has been reported by an increasing number of authors as summarized recently [23]. In a few patients, titration studies have shown a total absence of plasma α-gal A activity during agalsidase infusion suggesting that complete inactivation of the infused enzyme can occur in the presence of neutralizing antibody [26]. However, there have been limited publications on the clinical manifestations and therapeutic challenges of FD patients with high ADA titres and severe infusion-associated reactions (IARs). Here we report treatment challenges caused by the heightened immune response in two male adolescents who received ERT for up to three years.

## 2 Case presentations

Two male first cousins described below are part of a large kindred with FD. Upon confirming the FD diagnosis, both patients were enrolled in the CFDI study and the recommended schedule of baseline clinical assessments as per the CFDI protocol was followed [35]. The patients were both approved for ERT on the basis of neuropathic pain and gastrointestinal manifestations, unresponsive to standard medical management. They were subsequently randomized through the CFDI protocol to receive agalsidase beta. Of note these are the first males in this kindred to receive ERT. Their affected maternal uncle, now in his 40's, has classical disease with angiokeratomas, cornea verticillata, renal failure and cardiomyopathy but has declined any approved or experimental therapies. The mothers of these 2 cousins are confirmed manifesting carriers.

Patient 1 was diagnosed at 10 years of age. The diagnosis of FD had just been established in his maternal uncle. Patient 1 had long-standing intractable abdominal pain with nausea, cramping, diarrhea, constipation, and acroparesthesiae in his hands and feet dating back to early childhood. There was no symptomatic improvement with 6 months of treatment with ibuprofen, acetaminophen, and gabapentin. Intravenous ERT with agalsidase beta at recommended doses was initiated. While receiving ERT he required premedication with acetaminophen and antihistamines, as well as prolongation of infusion times to minimize IARs including fever and chills.

Patient 2 was diagnosed at 9 years of age after the diagnosis of FD was established in his maternal uncle. He had long-standing intractable abdominal pain and acroparesthesiae, as well as heat intolerance, headaches, and tinnitus. Symptoms were unchanged after 6 months of treatment with omeprazole, loperamide, acetaminophen, and ibuprofen. He thus was started on ERT with agalsidase beta at standard dose. He also required premedication with acetaminophen and antihistamines, as well as prolongation of infusion times to minimize IARs with fever, chills and abdominal pain.

After 3 years of ERT, with no improvement in symptoms of severe abdominal pain and acroparesthesiae, ERT was discontinued in both patients. They continue to experience neuropathic pain and GI symptoms. Cardiac and renal function remained normal during the 3 years of ERT and the subsequent 3 years as expected given their young age. Echocardiograms and cardiac magnetic resonance images have remained normal and there has been no evidence of proteinuria with normal eGFR.

## 3 Methods

Sanger sequencing of the *GLA* gene and α-gal A enzyme activity measured by standard fluorometric enzyme assay were performed by Mount Sinai Genetics Testing Laboratory (Dept. of Genetics and Genomic Sciences, Mount Sinai School of Medicine, New York, NY, USA).

IgG antidrug antibodies (ADA) against agalsidase beta were measured in serum samples by the enzyme linked immunosorbent assay (ELISA) (Genzyme Clinical Specialty Lab, Framingham, MA, USA), and IgE antibodies were measured by fluoroenzyme immunoassay (Genzyme Clinical Specialty Lab, Framingham, MA, USA) as previously described [7,39].

Serum samples were evaluated for the presence of anti-(α-Gal A) antibodies as previously described [38]. To assess neutralizing activity in vitro, serum (10 μL) was incubated with a standard amount of recombinant α-galactosidase A (agalsidase beta, 2.1 ng). Enzyme activity was determined after 10 min of incubation at room temperature. The serum dilution that resulted in 50% reduction of the enzyme activity was recorded (IC50). Titre is expressed as 1/x in which x is the dilution factor of serum. Appropriate controls were included and assays were done in triplicate.

Urinary Gb_3_ normalized to creatinine was analyzed according to the mass spectrophotometric method developed by Auray-Blais et al. [5]. Briefly, a homogenized urine specimen was deposited on a 10 × 10 cm filter paper (Whatman-GE 903) and dried at ambient air. The specimen was stored in a hermetic plastic bag at room temperature. For the analysis, a 5-cm filter paper disk was punched out and spiked with internal standards (100 μL of Gb_3_(C17:0) (1 μg/100 μL) and 100 μL of creatinine-D_3_ (40 μg/100 μL)). Thereafter, the paper disk was eluted with 4 mL of MeOH. The sample was separated by liquid chromatography using a Zorbax Bonus-RP Guard column (4.6 × 12.5 mm; Agilent Technologies, Santa Clara, CA, USA) and an Alliance 2795 HPLC system (Waters Corp., Milford, USA). The total of 8 Gb_3_ isoforms (C16:0, C18:0, C20:0, C22:1, C22:0, C24:1, C24:0, C24:OH), the Gb_3_(C17:0) internal standard, the creatinine and the creatinine-D_3_ internal standard were analyzed using the multiple reaction monitoring mode (MRM) with a Quattro Micro triple quadrupole mass spectrometer (Waters Corp.). Seven-point calibration curves were used for the quantitation of Gb_3_ (0–20 μg/mL) and creatinine (0–10 μmol/mL). Urine sample aliquots from an untreated Fabry male and an untreated Fabry female were used for the quality controls and analyzed with a tolerance of ±25%.

Analysis of plasma Lyso-Gb_3_ and analogues was conducted on plasma specimens collected in EDTA tubes and stored at -20 °C. The method developed by Boutin et al. [8,9], was used for the analysis of Lyso-Gb_3_ and analogues in plasma. A plasma volume of 100 μL was spiked with 500 μL of the glycinated Lyso-Gb_3_ standard (Lyso-Gb_3_-Gly, 10 nM in methanol). The samples were purified by solid phase extraction using a mixed-mode strong cation exchange (MCX) cartridge and separated by ultra-performance liquid chromatography (UPLC) using a BEH C_18_ column (1.7-μm, 2.1 × 50 mm, Waters Corp.) and an Acquity I-Class system (Waters Corp). Lyso-Gb_3_ and its 6 analogues (-C_2_H_4_, -H_2_, +O, +H_2_O, +H_2_O_2_, +H_2_O_3_), and the Lyso-Gb_3_-Gly internal standard were analyzed using the MRM mode with a Xevo TQ-S mass spectrometer (Waters Corp.). A seven-point calibration curve (0–400 nM) was used for the quantitation of Lyso-Gb_3_ and its analogues in plasma. Plasma sample aliquots from an untreated Fabry male and an untreated Fabry female were used for the quality controls and analyzed with a tolerance of ±20%.

The method developed by Lavoie et al. [21,22] was used for the analysis of Lyso-Gb_3_ and analogues in urine. Urine specimens were stored at -20 °C before analysis. A urine volume of 500 μL was spiked with 500 μL of the glycinated Lyso-Gb_3_ standard (Lyso-Gb_3_-Gly, 10 nM in methanol). The samples were purified by solid phase extraction using a MCX cartridge, and separated by ultra-performance liquid chromatography (UPLC) using an Atlantis HILIC Silica column (3 μm, 2.1 × 50 mm, Waters Corp.) and an Acquity I-Class system (Waters Corp). Lyso-Gb_3_ and its 7 analogues (-C_2_H_4_, -C_2_H_4_ + O, -H_2_, -H_2_ + O, +O, +H_2_O_2_, +H_2_O_3_), and the Lyso-Gb_3_-Gly internal standard were analyzed using the MRM mode with a Xevo TQ-S mass spectrometer (Waters Corp.). An eleven-point calibration curve (0–700 nM) was used for the quantitation of Lyso-Gb_3_ and its analogues in urine. The creatinine concentration was used to normalize the Lyso-Gb_3_ results [1]. Urine sample aliquots from an untreated Fabry male and an untreated Fabry female were used for the quality controls and analyzed with a tolerance of ±20%.

## 4 Results

The *GLA* gene consists of 7 exons and covers 12 kilobases (kb) of genomic DNA. Results of molecular testing in this family showed a 4.5 kb genomic *GLA* deletion, encompassing ~37% of the gene, in all affected family members tested. At the cDNA level, the deletion begins at 533 bases upstream from the first codon of *GLA* and extends to nucleotide 1277 (*GLA*c.370-533_c.1277del4.5).

Biochemical α-gal A enzyme activity testing in patient 1 revealed very low enzyme activity in leukocytes of 0.19 nmol/h/mg protein (normal range 12.8–74.1 nmol/h/mg protein) and plasma 0.30 nmol/h/mL (normal range 6.20–18.6 nmol/h/mL). In patient 2, results showed similarly very low α-galA enzyme activity in leukocytes 1.21 nmol/h/mg protein and in plasma 0.30 nmol/h/mL.

**Fig. 1:**
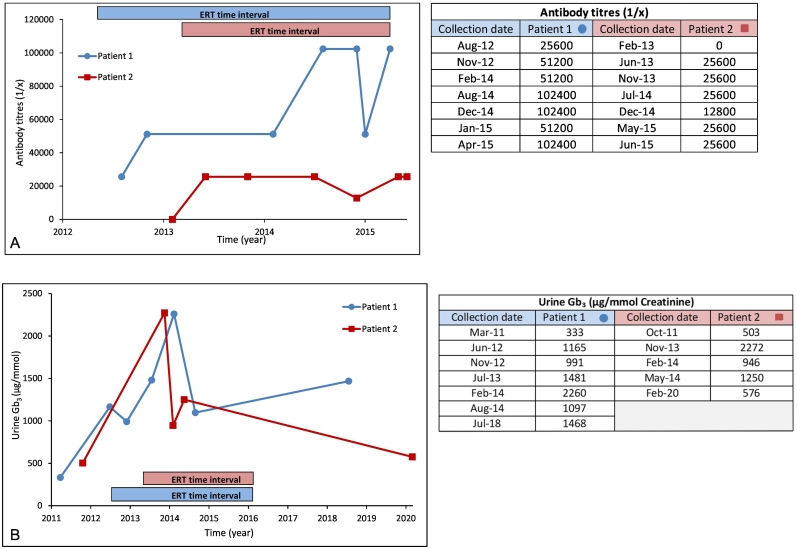
A. IgG antibody titres by ELISA assay for patients 1 and 2 in relation to ERT time course. B. Urine Gb_3_ levels for patients 1 and 2 in relation to ERT time course.

Results of serum IgG antibody titres for patients 1 and 2 in relation to ERT time course are plotted in Fig. 1A. For patient 1, no baseline antibody measurement was recorded prior to the start of ERT. IgG antibody titre was 1:25,600 (2 months after starting ERT) and rose to as high as 1:102,400. The results for IgE antibodies were consistently negative, meaning there was less than 0.35 k-units of IgE antibodies per litre of serum. There was one neutralizing antibody measurement performed after approximately 2.5 years of ERT. This showed that 1 mL of serum significantly inhibited 241 μg of enzyme in vitro. For patient 2, IgG antibodies were measured and not present at baseline prior to the start of ERT. At the highest, IgG antibody titre measured 1:25,600. IgE antibodies were consistently negative. Neutralizing antibody tests were performed on 2 occasions approximately 6 months apart. Successive results showed that 1 mL of serum inhibited 17 and 42 μg of enzyme activity in vitro.

**Fig. 2:**
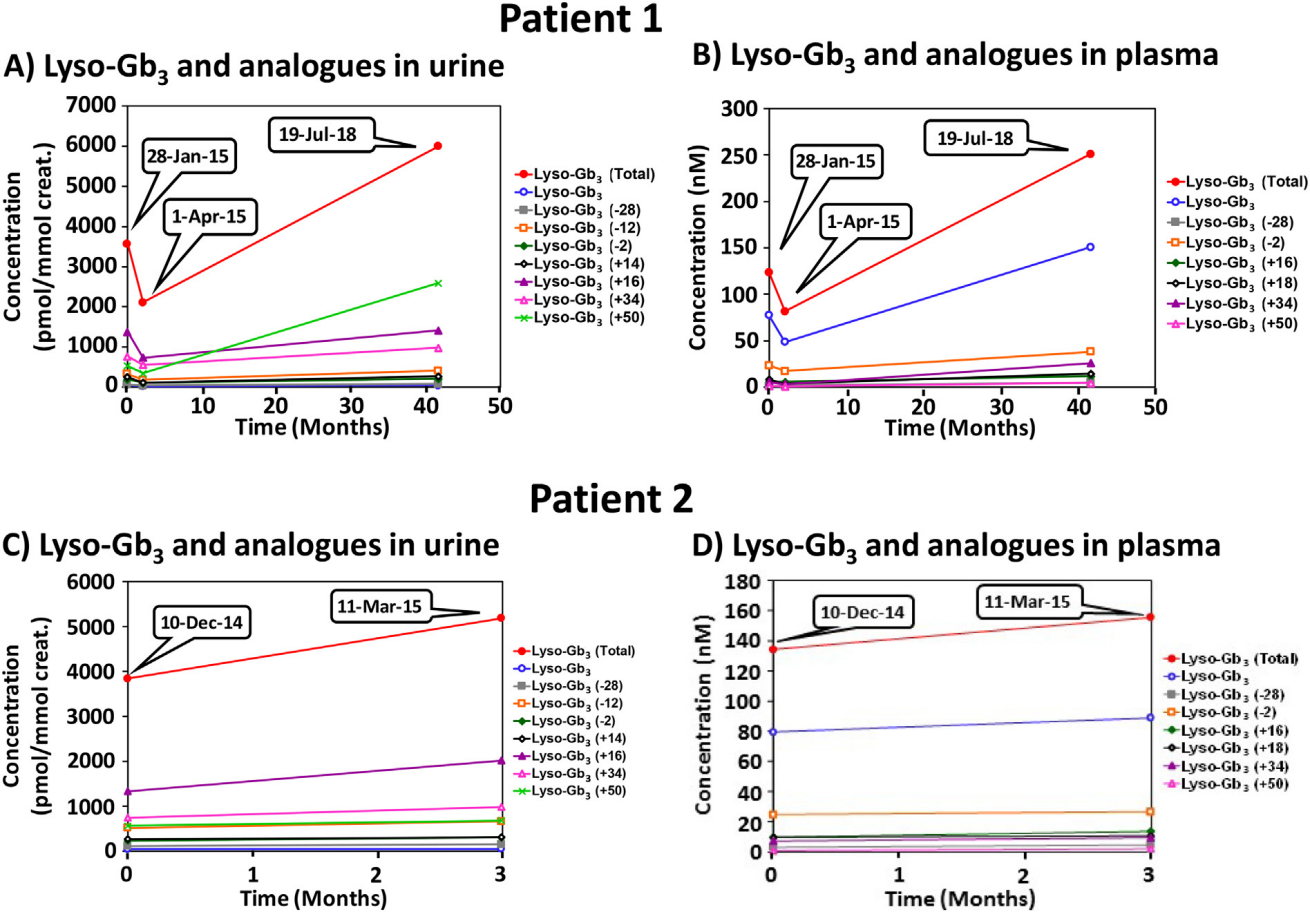
Longitudinal lyso-Gb_3_ and analogue biomarker variations in urine (panel A and C) and plasma (panels B and D) for patients 1 and 2, respectively. Patient 1 was on ERT with agalsidase-beta from June 2012 to April 2015. Patient 2 was also on ERT with agalsidase-beta from February 2013 to April 2015.

Results of urinary Gb_3_ for patients 1 and 2 in relation to ERT time course are plotted in Fig. 1B. For patient 1, urinary Gb_3_ showed an upward trend despite ERT from 333 to a peak of 2260 μg/mmol creatinine. Similarly, in patient 2, urinary Gb_3_ showed an upward trend from 503 to a peak of 1250 μg/mmol creatinine. In patient 1, plasma and urinary Lyso-Gb_3_ measured 77.3 nmol/L (normal value is 2.4 nmol/L) and 50 pmol/mmol creatinine respectively (normal value is “not detected”) (Fig. 2 A and B). In patient 2, plasma and urinary Lyso-Gb_3_ were measured as high as 79.5 nmol/L and 56 pmol/mmol creatinine, respectively (Fig. 2 C and D) (See Supplemental Table 1 for detailed Lyso-Gb_3_ values).

## 5 Discussion

This report documents the persisting very high ADA response to ERT in two male adolescent FD patients receiving ERT with standard dose agalsidase beta.

A key concern in the first FD ERT trials was immediate IARs. IARs were originally felt to be IgE mediated but are now known to be “anaphylactoid” (compound mediated) and IgG mediated consistent with the fact that both our patients had persisting severe IARs but remained IgE negative. Premedication protocols, usually a combination of single dose acetaminophen or a non-steroidal anti-inflammatory drug, antihistamine and corticosteroid, along with increasing infusion time, to prevent such reactions have been established successfully and are used as required [13]. While receiving ERT at recommended doses, both patients required premedication which attenuated, but did not prevent, IARs.

To date, there remains no common ADA assay used in the care of FD patients. Assays are done via various techniques (ELISA, in vitro inhibition) and differ in their sensitivities and specificities which make comparisons difficult. An attempt to develop a common assay has failed (Schellenkens et al. 2008). Seroconversion with ADA is reported in 73% males as opposed to 12% females treated with agalsidase beta. This is likely due to complete absence of native enzyme in males with classical FD while males with missense mutations, who express residual α-Gal A enzyme activity, are far less likely to develop antibodies [39]. Neutralizing antibodies detected in vitro may not correlate with in vivo action. However, as identified by the method of Aerts, these antibodies cross react with both agalsidase products, tend to be persistent and have only been reported in males with FD [30]. Long-term effect of neutralizing antibodies has been studied in detail, in particular, their impact on clinical outcomes. Bénichou et al. [7] observed significantly impaired Gb_3_ clearance in skin biopsies of patients treated with ERT and high antibody titres. A correlation between ADA levels and plasma Lyso-Gb_3_ has been confirmed [31]. The same has been reported for ADA levels and urine Gb_3_ [30]. Similarly, when analyzing in vitro enzyme inhibition in relation to plasma Lyso-Gb_3_ levels, disease severity scores and subjective symptoms in a group of 168 patients, Lenders et al. [24] confirmed higher Lyso-Gb_3_ levels, as well as worsening disease severity scores in patients with serum-mediated enzyme inhibition. As well, results reported by Auray-Blais et al. [3,4] from a large cohort of FD patients in Taipei revealed that plasma and urine Lyso-Gb_3_ analogues (+16), (+34), and (+50) were positively associated with the severity of disease for the left ventricular mass index and also the Mainz Severity Score Index when adjusted for age and gender. High levels of these glycosphingolipids were also identified in children. Moreover, and similar to the results obtained for patients 1 and 2 where the analogues of Lyso-Gb_3_ in urine are markedly increased, a study in FD children showed that the urine Lyso-Gb_3_ analogues are sometimes increased greater than Lyso-Gb_3_ itself [2]. Sakuraba et al. [31] showed that there was no difference noted in ADA with respect to type of ERT administered (agalsidase alfa or beta) and did not recommend a therapy switch in their antibody-positive patients.

The aim of ERT is to minimize FD symptoms, prevent disease progression and Gb_3_ and Lyso-Gb_3_ build up. We had previously measured the levels of urine Gb_3_ in “liquid” urine specimens from 33 male FD patients receiving ERT and the results ranged from 0.45 μg/mmol creatinine to 129 μg/mmol creatinine [10]. These values were obtained after conversion of the units from nM/mM creatinine to μg/mmol creatinine. Only the 8 Gb_3_ isoforms analyzed on filter paper (C16:0, C18:0, C20:0, C22:1, C22:0, C24:1. C24:0, C24:OH) were taken in account for the unit conversion, and the outlier values (over 1.5 times the interquartile range) were excluded.

In both of our patients reported herein, urine Gb_3_ levels greatly exceeded this range while receiving ERT suggesting a blunting or lack of effectiveness of ERT in these 2 patients (Fig. 1B). Levels of Lyso-Gb_3_ and analogues in urine and plasma were also massively and persistently elevated consistent with the suggested loss of ERT action. Lyso-Gb_3_ levels increased up to 46 and 40 times the normal values in urine, and up to 70 and 43 times the normal values in plasma for patients 1 and 2, respectively (Fig. 2).

The mechanisms by which neutralizing ADA can interfere with ERT in FD have not been well studied. Lenders et al. [26] showed a reduction in plasma agalsidase activity during ERT infusion in Fabry patients with neutralizing ADA consistent with direct antibody binding of the enzyme. Three patients treated with Pegunigalsidase developed neutralizing ADA with transient altered drug pharmacokinetics with lower area under the concentration curve, lower maximum observed concentration and longer terminal half-life [34] also consistent with direct ADA binding and inactivation of the infused enzyme. Other mechanisms such as ADA interference with enzyme uptake into cells and increased enzyme clearance are possible but have not been studied in ADA in Fabry disease.

To overcome the ADA effect, immunomodulation therapy in response to antibody formation has been initiated in other LSDs treated with ERT [11]. Immunomodulation therapy has had documented success in both Gaucher disease [12] and Pompe disease [29] and this has enabled patients to continue ERT. Successful immunomodulation has also been demonstrated in FD mouse models [15]. A study investigating the effect of immunosuppression in renal transplant patients with FD on antibody formation has provided valuable insights [25]. Immunosuppression was shown to decrease ADAs significantly in ERT naive as well as treated patients. However, further studies are needed to evaluate the clinical impact of lowering ADAs and its correlation with biomarkers and clinical response. Patients with persistent very high antibody titres may not respond to immunomodulation therapy [6].

Due to the demanding nature of an immunomodulation regime, its potential side effects, and uncertain outcomes, the patients and their families declined this approach. Immune tolerance induction through dose escalation was considered as another alternative. However, this option was also declined as impractical to due high cost. Rombach et al. [30] reported that increased ERT dose by switching from agalsidase alfa 0.2 mg/kg to agalsidase beta 1.0 mg/kg in Fabry patients with ADA resulted in lower plasma levels of both Lyso-Gb_3_ and Gb_3_. However, Lenders et al. [26] described a small number of patients with neutralizing antibody in whom the response to ERT dose increase was quite variable; some patients had no response or only a transient response in the short term as determined by the calculated plasma agalsidase activity by titration during ERT infusion. Further study is needed to define the response to increased enzyme dosing in the presence of neutralizing ADA. An alternate ERT formulation was considered, however agalsidase alfa is structurally similar to agalsidase beta so similar effects would very likely be elicited due to the patients' preexisting immune response. Several studies have found that all antibodies showed complete cross-reactivity such that patients who developed antibodies after treatment with agalsidase alfa or beta had the same antibody titre toward the other [27,31]. Chaperone therapy and substrate reduction therapy (SRT) are newer therapeutic options that were also considered. However, the patients' mutations are not amenable to the oral chaperone therapy option migalastat [17] leaving only SRT as an option. The SRT lucerastat (Idorsia Pharmaceuticals Ltd., Allschwil, Switzerland) is currently in a clinical trial phase but only adults who meet entry criteria are presently eligible [18]. This made both patients who are minors still ineligible for this SRT trial. Gene therapy is another option that could be considered in the future when the patients reach adulthood [19]. Such gene therapy trials are currently underway in Canada [28]. However, pre-existing ADA could in theory also interfere with agalsidase production by transfected cells and little is known currently about ADA in gene therapy in lysosomal diseases.

In conclusion, high ADA titres can pose a significant treatment barrier for FD. We suggest that the recognition that a male FD patient is not responding well to ERT is reason to look for the presence of high titre neutralizing ADA that may be limiting the action of infused enzyme.

Routine periodic antibody screening in patients treated with ERT, as well as markedly increased biomarker levels in biological fluids, will allow early detection of neutralizing ADA and could permit early ERT dose increase, immunomodulation treatment or a switch to a different therapy option when it is possible. To date, there is no suitable treatment available for our young patients. The patients presented here illustrate the need for more research into other therapeutic options, such as SRT, new chaperone therapies for mutations that are not amenable to migalastat, or other novel treatment approaches for ADA reduction and prevention to improve the efficacy of ERT (Stappers et al. 2020) [37], as well as gene therapy.

## Appendix A Supplementary data

Supplementary material Image 1
